# Analysis and Improvement of Indoor Positioning Accuracy for UWB Sensors

**DOI:** 10.3390/s21175731

**Published:** 2021-08-25

**Authors:** Leehter Yao, Lei Yao, Yeong-Wei Wu

**Affiliations:** Department of Electrical Engineering, National Taipei University of Technology, Taipei 10608, Taiwan; arleyyao@gmail.com (L.Y.); andywu8491@yahoo.com (Y.-W.W.)

**Keywords:** UWB, positioning accuracy, distance measurement, time of flight, Gaussian distribution

## Abstract

Ultra-wideband (UWB) sensors have been widely applied to indoor positioning. The indoor positioning of UWB sensors usually refers to the positioning of the mobile node that interacts with the anchors through radio for calculating the distance between the mobile node and each of the surrounding anchors. The positioning accuracy of the mobile node is affected by the installation positions of surrounding anchors. A mathematical model was proposed in this paper to respectively analyze the mobile node’s 2-dimensional (2D) and 3-dimensional (3D) positioning errors. The factors influencing the mobile node’s positioning errors were explored through the mathematical models. The best installation positions of surrounding anchors were obtained based on the mathematical models. The mobile node’s 2D and 3D positioning errors were reduced based on the anchor positions derived from the mathematical model. Both computer simulations and practical experiments were implemented to justify the results obtained in the mathematical models.

## 1. Introduction

The Global Positioning System (GPS) is widely used for outdoor positioning and navigation. The GPS receivers receive the GPS signals transmitted from the satellite system for positioning. The received GPS signals are utilized to calculate the user’s 3-dimensional (3D) position. However, the GPS signals transmitted from the satellites are easily blocked by building structures, including roofs, floors, walls, etc. Indoor positioning is not feasible using GPS signals. With the increasing need for indoor positioning for customers in shopping malls, patients in hospitals, containers in terminals, and materials in warehouses [1,2,3,4], the indoor positioning technology industry is growing rapidly. Various indoor positioning systems are already in the market, including ultrasound [5], infrared [6], Bluetooth [7], Zigbee [8], RFID [9], and Wi-Fi [10], and each has its advantages and disadvantages. Ultrasound is susceptible to multipath effects, and its detection range is limited by distance, angle, and obstruction. Infrared is vulnerable to light and obstacles. Bluetooth, Zigbee, and Wi-Fi are based on a received signal strength indicator (RSSI) [8] and are severely affected by barriers, resulting in extensive positioning errors. In addition, RFID is a short-range wireless communication technology; therefore, it is limited by distance. The ultra-wideband (UWB) sensor proposed herein provides reliable, high-precision ranging, covers a relatively wide area, and can bypass certain obstacles [11]. The proposed UWB positioning system comprises multiple anchors and mobile nodes; the measured distances between the anchors and the mobile node are used for positioning.

Localization methods for UWB sensors include trilateration [12], the least-squares (LS) method [13], partial filtering [14], and the extended Kalman filter (EKF) [15]. EKF outperforms other methods regarding positioning accuracy and stability, particularly when the UWB-sensor data have outliers. To further improve positioning accuracy, the UWB mobile node is integrated with an inertia measurement unit (IMU) [16,17,18,19,20,21] to eliminate outliers in the received data. UWB mobile node measures the values such as angle of arrival (AOA) [22], time of arrival (TOA) [23], time of flight (TOF), and time differential of arrival (TDOA) [14], etc., to calculate the position of a mobile node. TOA, TOF, and AOA are based on radio transmission and receiving time differences, which can be used to measure the distance and directional angle. TDOA determines the position of the mobile node based on the time difference between the signals received by different anchors.

Numerous methods have been employed to improve UWB’s positioning accuracy. Multi-sensor fusion is effective for achieving high-precision positioning [24,25,26,27,28]. Combining UWB positioning with an IMU can eliminate drift-free output in UWB-sensor data and correct accumulated IMU errors [29,30]. Both Light-of-sight (LOS) and non-light-of-sight (NLOS) analysis methods can improve the stability and accuracy of UWB positioning systems [31,32,33]. The position accuracy of the mobile node is affected by the installation positions of anchors. The anchors are not all placed in a plane to reduce the mobile node’s 3D position accuracy [14,34,35]. Experimental results in [34] indicated that positioning accuracy differs in the z-axis at different anchor heights, but the reasons were not thoroughly explained. The UWB positioning error was investigated in [36] by using the concept of dilution of precision [37]. A 2-dimensional (2D) positioning method was proposed for automated guided vehicles (AGVs) in [38] to investigate the effects of the distance between neighboring anchors on the positioning accuracy. The mathematical model estimating positioning errors proposed in [38] was specifically for the environment, such as the corridor where the anchors are installed on the wall of the corridor. The heights (positions on the z-axis) of all the anchors on the wall were assumed to be the same. No constraints such as in [38] have been imposed on the installation positions of anchors. In other words, the anchors are not limited to be installed on the wall of a corridor, and the anchors are not necessarily to be installed at the same height on the wall. The mathematical model proposed in [38] was only for calculating the 2D positions of the mobile node. In contrast to the model proposed in [36], the model presented in this paper can be utilized to calculate both 2D and 3D positions. There is no other suitable mathematical model for UWB sensors estimating mobile node’s 3D positioning errors to our best knowledge.

The positioning accuracy of UWB sensors refers to the positioning accuracy of the mobile node. The mobile node’s 2D or 3D position is calculated based on the distance between the mobile node and its surrounding anchors. Therefore, UWB positioning accuracy depends on the installation positions of surrounding anchors. It takes at least three and four anchors for 2D and 3D positioning, respectively. Mathematical models are proposed to analyze both the 2D and 3D positioning accuracies of the UWB mobile node for both the LOS and NLOS conditions. The variance of 2D and 3D positioning errors are mathematically derived. Technically speaking, the mathematical model of 2D positioning errors is a special case of 3D. The best arrangement of anchor installation positions reducing the positioning errors is obtained based on the mathematical model of positioning errors. Computer simulations and practical experiments are then designed to verify anchor installation positions obtained from the mathematical model. The root-mean-square positioning error (RMSPE) of the mobile node is calculated in different arrangements of anchor installation positions. It is shown that both the 2D and 3D RMSPE are reduced if the anchors are installed at the positions suggested in the mathematical model of positioning errors.

The main contributions of this paper are as follows:

- Mathematical models of both 2D and 3D positioning errors for UWB sensors were derived. To the best of our knowledge, this paper is the first one analyzing suitable UWB anchor installation positions based on a mathematical model of 3D positioning errors.
- The mathematical models of 2D and 3D positioning errors impose no constraints on anchors’ installation positions. The models are general enough to analyze mobile node’s positioning errors corresponding to any anchor installation positions.
- Anchor installation positions were suggested based on the mathematical model of 2D and 3D positioning errors for both LOS and NLOS conditions so that the RMSPE can be significantly reduced.
- Both computer simulations and practical experiments were conducted to verify that the anchor installation positions suggested based on the mathematical model of positioning errors can significantly reduce the RMSPE.

The remainder of the paper is organized as follows. The problem statements and indoor positioning system with UWB sensors are described in Section 2. The mathematical models of 2D and 3D positioning errors are derived in Section 3. The factors affecting the mobile node’s position accuracy are also analyzed. The computer simulations using MATLAB are designed in Section 4 to justify that the anchor installation positions suggested based on the mathematical model do significantly reduce the positioning error. Several practical experiments are further conducted in Section 5 to explain the results obtained through computer simulations practically. Finally, the conclusions are drawn in Section 6. 

## 2. Problem Statement

### System Design

**Figure 1:**
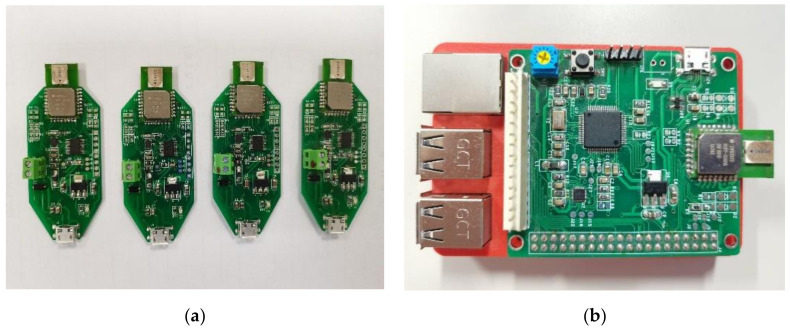
The UWB sensors used in this paper. (**a**) anchors; (**b**) mobile node.

The indoor positioning system utilized in this paper was implemented using UWB sensors DecaWave DWM1000 [39], with an optional frequency band range from 3.5 to 6.5 GHz. The data transmission rates included 110 kbps, 850 kbps, and 6.8 Mbps. The anchor was implemented as in Figure 1a using an STM32F0 microcontroller unit (MCU) with the main frequency of 48 MHz and a UWB sensor DWM1000. The mobile node was implemented as in Figure 1b, combining an STM32F4 MCU with the main frequency of 168 MHz, an IMU MPU 9250, and a UWB sensor DWM1000. An embedded system made with a Raspberry Pi 3 was utilized in the mobile node for positioning calculation and the implementation of EKF to improve positioning results. The sampling frequency for positioning was set as 100 Hz. 

The positioning of the mobile node was based on measuring the distance between the mobile node and every anchor. The distance measurement relied on the TOF of the radio between the transmitter and the receiver. Two-way radio transmission and receiving were conducted between both sensors. The mobile node was designated as an initiator that initiates the two-way radio communication, while the anchor was set as a responder. The initiator transmits a message through radio to the responder and records the timestamp of transmission. As the responder receives the message, it sends the same message back to the initiator after a preset time delay. The initiator gets the message and records the timestamp of receiving. The TOF is calculated based on the timestamps of transmission and receiving, and the preset time delays at both the responder and the initiator. The calculation of TOF is affected by the clock drift, frequency drift, fading, shadowing, multi-path propagation, etc. Although more delicate ranging methods such as the asymmetric double-sided two-way-ranging (TWR) method [39] or the TWR method integrated with neural network model [40] have been proposed, noise in the measurement of TOF is unavoidable. Let $T_{tof}$ and ${\hat{T}}_{tof}$ be the ideal TOF and the measured TOF, respectively, and $\delta T_{tof}$ be the noise in TOF measurement. Then,

(1) $${\hat{T}}_{tof}=T_{tof}+\delta T_{tof}.$$

The measured distance $r_{m}$ between the mobile node and the anchor is calculated as

(2) $$r_{m}=c(T_{tof}+\delta T_{tof})=r+\varepsilon ,$$

where *c* is the speed of radio wave, *r* is the ideal distance between the mobile node and the anchor, and ε is the noise of measured distance due to the noise of TOF, $\delta T_{tof}$. The noise ε in (2) is usually modeled as an independent and identical distributed (i.i.d.) random variable. The distribution of the noise ε is usually modeled as the Gaussian distribution [41,42] if the mobile node and the anchor are in the LOS condition. The Gaussian distribution is parameterized with the mean $\mu_{G}$ and the variance $\sigma_{G}{}^{2}$, i.e., $\varepsilon ∼N\left(\mu_{G},\sigma_{G}^{2}\right)$. The noise modeled in the LOS condition is mainly for the environment that no obstacles are placed to block the radio propagation between the mobile node and the corresponding anchor. 

The distribution of the noise ε is modeled as the skew-t distribution [43] for the NLOS condition. The skew-t distribution is parameterized by its location parameter $\mu_{ST}$, spread parameter $\sigma_{ST}^{2}$, shape parameter $\delta_{ST}$, and degree of freedom $\nu_{ST}$. The probability density function (PDF) of the skew-t distribution for the random variable *z* is defined as

(3) $$ST(z;\mu_{ST},\sigma_{ST}^{2},\delta_{ST},\nu_{ST})=2t(z;\mu_{ST},\delta_{ST}^{2}+\sigma_{ST}^{2},\nu_{ST})B(\tilde{z};0,1,\nu_{ST}+1),$$

where t(·) denotes the PDF of Student’s t-distribution defined as follows:

(4) $$t(z;\mu_{ST},\sigma_{ST}^{2},\nu_{ST})=\frac{\Gamma (\frac{\nu_{ST}+1}{2})}{\sigma_{ST}\sqrt{\nu_{ST}\pi }\Gamma (\frac{\nu_{ST}}{2})}{\left(1+\frac{{\left(z-\mu_{ST}\right)}^{2}}{\nu_{ST}\sigma_{ST}^{2}}\right)}^{-\frac{\nu_{ST}+1}{2}}.$$

Note that Γ(·) in (4) denotes the Gamma function. The function B(·) in (3) denotes the cumulative distribution function (CDF) of Student’s t-distribution and the random variable $\tilde{z}$ in B is defined as:

(5) $$\tilde{z}=\frac{(z-\mu_{ST})\delta_{ST}}{\sigma_{ST}}\sqrt{\frac{\nu_{ST}+1}{\nu_{ST}(\delta_{ST}^{2}+\sigma_{ST}^{2})+{\left(z-\mu_{ST}\right)}^{2}}}.$$

Therefore, the distance measurement noise $\varepsilon ∼ST(z;\mu_{ST},\sigma_{ST}^{2},\delta_{ST},\nu_{ST})$ for the NLOS condition where skew t-distribution ST(·) is defined in (3). The noise modeled in the NLOS condition is mainly for the environment that obstacles are placed between the mobile node and the anchor so that the LOS radio propagation is blocked. The radio multi-path propagation due to moving and/or stationary obstacles usually result in the noise modeled in the NLOS condition [44]. 

## 3. Mathematical Model of Positioning Errors

### 3.1. 3D Positioning

**Figure 2:**
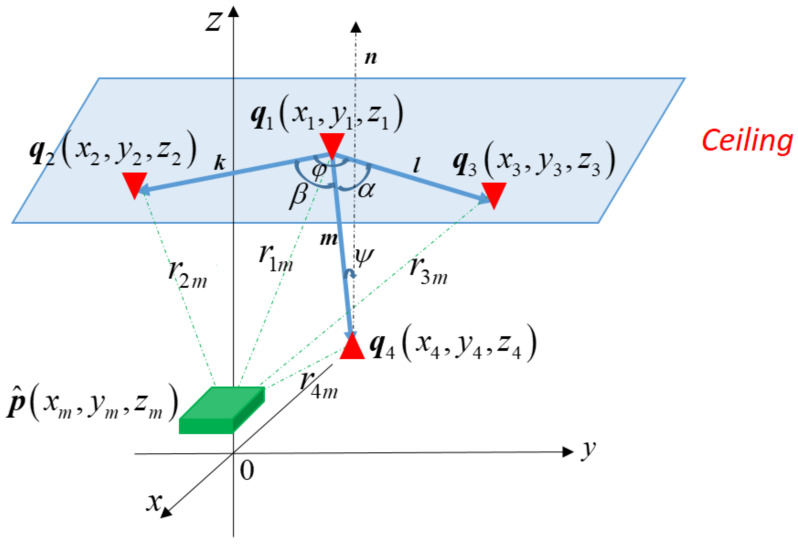
Positioning of a mobile node in a 3D space with 4 anchors.

At least four anchors are required for 3D positioning of a mobile node, as shown in Figure 2. Denote $q_{i}$ as the vector containing the coordinate $\left(x_{i},y_{i},z_{i}\right)$ of the *i*th anchors, i.e., $q_{i}={\left[x_{i},y_{i},z_{i}\right]}^{T}$, *i* = 1, …,4. The 3D position of the mobile node can be determined by first measuring the distance between the mobile node and every anchor. An EKF is utilized to calculate the position of the mobile node. Denote ***p*** and $\hat{p}$ as the vector containing the coordinate the mobile node’s actual 3D position (*x, y, z*) and measured 3D position $\left(x_{m},y_{m},z_{m}\right)$, respectively, i.e., $p={\left[x,y,z\right]}^{T}$ and $\hat{p}={\left[x_{m},y_{m},z_{m}\right]}^{T}$. The measured distance between the mobile node and the *i*th anchor is calculated as

(6) $$r_{im}=\sqrt{{\left(x_{m}-x_{i}\right)}^{2}+{\left(y_{m}-y_{i}\right)}^{2}+{\left(z_{m}-z_{i}\right)}^{2}},\;i=1,\;\ldots ,4.$$

Similarly, the actual distance between the mobile node and the *i*th anchor is calculated as

(7) $$r_{i}=\sqrt{{\left(x-x_{i}\right)}^{2}+{\left(y-y_{i}\right)}^{2}+{\left(z-z_{i}\right)}^{2}},\;i=1,\;\ldots ,4.$$

Referring to (2), the measured distance can be represented as

(8) $$r_{im}=r_{i}+\varepsilon_{i},\;i=1,\;\ldots ,4.$$

where $\varepsilon_{i}$ denotes the measurement noise of the distance between the mobile node and the *i*th anchor. 

Referring to Figure 2, let $k={\left[x_{2}-x_{1},y_{2}-y_{1},z_{2}-z_{1}\right]}^{T}$, $l={\left[x_{3}-x_{1},y_{3}-y_{1},z_{3}-z_{1}\right]}^{T}$, and $m={\left[x_{4}-x_{1},y_{4}-y_{1},z_{4}-z_{1}\right]}^{T}$ be the vectors from the position $q_{1}$ to $q_{2}$, $q_{1}$ to $q_{3}$, and $q_{1}$, to $q_{4}$, respectively. Note that three points at different places form a plane. For the convenience of analysis, let the vectors ***k*** and ***l*** be on the same plane.

Let n=k×l, d=l×m, and g=k×m, where

(9) $$n=\left[\begin{array}{c} \left(y_{2}-y_{1})(z_{3}-z_{1})-(z_{2}-z_{1})(y_{3}-y_{1}\right)\\ -(x_{2}-x_{1})(z_{3}-z_{1})+(z_{2}-z_{1})(x_{3}-x_{1})\\ \left(x_{2}-x_{1})(y_{3}-y_{1})-(y_{2}-y_{1})(x_{3}-x_{1}\right) \end{array}\right],$$

(10) $$d=\left[\begin{array}{c} \left(y_{4}-y_{1})(z_{3}-z_{1})-(z_{4}-z_{1})(y_{3}-y_{1}\right)\\ -(x_{4}-x_{1})(z_{3}-z_{1})+(z_{4}-z_{1})(x_{3}-x_{1})\\ \left(x_{4}-x_{1})(y_{3}-y_{1})-(y_{4}-y_{1})(x_{3}-x_{1}\right) \end{array}\right],$$

(11) $$g=\left[\begin{array}{c} \left(y_{4}-y_{1})(z_{2}-z_{1})-(z_{4}-z_{1})(y_{2}-y_{1}\right)\\ -(x_{4}-x_{1})(z_{2}-z_{1})+(z_{4}-z_{1})(x_{2}-x_{1})\\ \left(x_{4}-x_{1})(y_{2}-y_{1})-(y_{4}-y_{1})(x_{2}-x_{1}\right) \end{array}\right].$$

With the measured distance $r_{im}$ defined in (6),

(12) $$\left[\begin{array}{c} \left(r_{1m}^{2}-r_{2m}^{2}\right)\\ \left(r_{1m}^{2}-r_{3m}^{2}\right)\\ \left(r_{1m}^{2}-r_{4m}^{2}\right) \end{array}\right]=\left[\begin{array}{c} 2\left(x_{2}-x_{1}\right)x_{m}+2\left(y_{2}-y_{1}\right)y_{m}+2\left(z_{2}-z_{1}\right)z_{m}+\left(x_{1}^{2}-x_{2}^{2}\right)+\left(y_{1}^{2}-y_{2}^{2}\right)+\left(z_{1}^{2}-z_{2}^{2}\right)\\ 2\left(x_{3}-x_{1}\right)x_{m}+2\left(y_{3}-y_{1}\right)y_{m}+2\left(z_{3}-z_{1}\right)z_{m}+\left(x_{1}^{2}-x_{3}^{2}\right)+\left(y_{1}^{2}-y_{3}^{2}\right)+\left(z_{1}^{2}-z_{3}^{2}\right)\\ 2\left(x_{4}-x_{1}\right)x_{m}+2\left(y_{4}-y_{1}\right)y_{m}+2\left(z_{4}-z_{1}\right)z_{m}+\left(x_{1}^{2}-x_{4}^{2}\right)+\left(y_{1}^{2}-y_{4}^{2}\right)+\left(z_{1}^{2}-z_{4}^{2}\right) \end{array}\right].$$

Therefore,

(13) $$\left[\begin{array}{c} \left(r_{1m}^{2}-r_{2m}^{2})+(x_{2}^{2}-x_{1}^{2})+(y_{2}^{2}-y_{1}^{2})+(z_{2}^{2}-z_{1}^{2}\right)\\ \begin{array}{l} \left(r_{1m}^{2}-r_{3m}^{2})+(x_{3}^{2}-x_{1}^{2})+(y_{3}^{2}-y_{1}^{2})+(z_{3}^{2}-z_{1}^{2}\right)\\ \left(r_{1m}^{2}-r_{4m}^{2})+(x_{4}^{2}-x_{1}^{2})+(y_{4}^{2}-y_{1}^{2})+(z_{4}^{2}-z_{1}^{2}\right) \end{array} \end{array}\right]=2\left[\begin{array}{l} \left(x_{2}-x_{1}\right)\left(y_{2}-y_{1}\right)\left(z_{2}-z_{1}\right)\\ \left(x_{3}-x_{1}\right)\left(y_{3}-y_{1}\right)\left(z_{3}-z_{1}\right)\\ \left(x_{4}-x_{1}\right)\left(y_{4}-y_{1}\right)\left(z_{4}-z_{1}\right) \end{array}\right]\left[\begin{array}{c} x_{m}\\ y_{m}\\ z_{m} \end{array}\right].$$

Referring to (13), let

(14) $$\Delta r_{m}=\left[\begin{array}{c} \left(r_{1m}^{2}-r_{2m}^{2})+(x_{2}^{2}-x_{1}^{2})+(y_{2}^{2}-y_{1}^{2})+(z_{2}^{2}-z_{1}^{2}\right)\\ \begin{array}{l} \left(r_{1m}^{2}-r_{3m}^{2})+(x_{3}^{2}-x_{1}^{2})+(y_{3}^{2}-y_{1}^{2})+(z_{3}^{2}-z_{1}^{2}\right)\\ \left(r_{1m}^{2}-r_{4m}^{2})+(x_{4}^{2}-x_{1}^{2})+(y_{4}^{2}-y_{1}^{2})+(z_{4}^{2}-z_{1}^{2}\right) \end{array} \end{array}\right],$$

and

(15) $$H=\left[\begin{array}{l} \left(x_{2}-x_{1}\right)\left(y_{2}-y_{1}\right)\left(z_{2}-z_{1}\right)\\ \left(x_{3}-x_{1}\right)\left(y_{3}-y_{1}\right)\left(z_{3}-z_{1}\right)\\ \left(x_{4}-x_{1}\right)\left(y_{4}-y_{1}\right)\left(z_{4}-z_{1}\right) \end{array}\right]\;.$$

Then, (13) is expressed as

(16) $$\Delta r_{m}\;=2H\hat{p},$$

where $\hat{p}$ is the measured 3D position of the mobile node, $\hat{p}={\left[x_{m},y_{m},z_{m}\right]}^{T}$. If ***H*** is nonsingular, i.e., det(***H***) *≠ 0*, the measured position of the mobile node can be calculated as

(17) $$\hat{p}=\frac{1}{2}H^{-1}\Delta r_{m}.$$

Since n=k×l, *n* is perpendicular to the plane formed by three anchor positions $q_{1},q_{2}\;\mathrm{and}\;q_{3}$. Referring to (14), $\hat{p}$ is calculated under the condition that det(*H*) *≠ 0*. However, $\det\left(H\right)=n^{T}m$, the condition det(***H***) *≠ 0* results in the condition that ***m*** is not perpendicular to the vector ***n***, i.e., ***m*** is not on the plane formed by three anchor positions $q_{1},q_{2}\;\mathrm{and}\;q_{3}$. In other words, the fourth anchor position $q_{4}$ cannot be on the same plane with $q_{1},q_{2}\;\mathrm{and}\;q_{3}$ in order to have a deterministic position $\hat{p}$ for the mobile node. 

Substituting the actual distance $r_{i}$ between the mobile node to the *i*th anchor into (12)–(14), the actual position ***p*** associated with the actual distance can be theoretically determined similar to the measured position in (17) as

(18) $$p=\frac{1}{2}H^{-1}\Delta r,$$

where

(19) $$\Delta r=\left[\begin{array}{c} \left(r_{1}^{2}-r_{2}^{2})+(x_{2}^{2}-x_{1}^{2})+(y_{2}^{2}-y_{1}^{2})+(z_{2}^{2}-z_{1}^{2}\right)\\ \begin{array}{l} \left(r_{1}^{2}-r_{3}^{2})+(x_{3}^{2}-x_{1}^{2})+(y_{3}^{2}-y_{1}^{2})+(z_{3}^{2}-z_{1}^{2}\right)\\ \left(r_{1}^{2}-r_{4}^{2})+(x_{4}^{2}-x_{1}^{2})+(y_{4}^{2}-y_{1}^{2})+(z_{4}^{2}-z_{1}^{2}\right) \end{array} \end{array}\right].$$

Denote Δp as the mobile node’s positioning error,

(20) $$\Delta p\equiv p-\hat{p}=\frac{1}{2}H^{-1}\left(\Delta r-\Delta r_{m}\right)=\frac{1}{2}H^{-1}\Delta \lambda .$$

where $\Delta \lambda =\Delta r-\Delta r_{m}$. Referring to (14) and (19), Δλ is calculated as

(21) $$\Delta \lambda =\left[\begin{array}{c} 2r_{2}\varepsilon_{2}-2r_{1}\varepsilon_{1}+\varepsilon_{2}^{2}-\varepsilon_{1}^{2}\\ \begin{array}{l} 2r_{3}\varepsilon_{3}-2r_{1}\varepsilon_{1}+\varepsilon_{3}^{2}-\varepsilon_{1}^{2}\\ 2r_{4}\varepsilon_{4}-2r_{1}\varepsilon_{1}+\varepsilon_{4}^{2}-\varepsilon_{1}^{2} \end{array} \end{array}\right].$$

Substituting (21) into (20) and going through some derivations yields

(22) $$\Delta p=\frac{a_{2}M_{3}m+a_{3}M_{2}m+a_{4}n}{n^{T}m},$$

where

(23) $$M_{2}=\left[\begin{array}{ccc} 0 & z_{2}-z_{1} & -(y_{2}-y_{1})\\ -(z_{2}-z_{1}) & 0 & x_{2}-x_{1}\\ y_{2}-y_{1} & -(x_{2}-x_{1}) & 0 \end{array}\right],$$

(24) $$M_{3}=\left[\begin{array}{ccc} 0 & -(z_{3}-z_{1}) & y_{3}-y_{1}\\ z_{3}-z_{1} & 0 & -(x_{3}-x_{1})\\ -(y_{3}-y_{1}) & x_{3}-x_{1} & 0 \end{array}\right],$$

(25) $$a_{2}=\frac{1}{2}\left(2r_{2}\varepsilon_{2}-2r_{1}\varepsilon_{1}+\varepsilon_{2}^{2}-\varepsilon_{1}^{2}\right),$$

(26) $$a_{3}=\frac{1}{2}\left(2r_{3}\varepsilon_{3}-2r_{1}\varepsilon_{1}+\varepsilon_{3}^{2}-\varepsilon_{1}^{2}\right),$$

(27) $$a_{4}=\frac{1}{2}\left(2r_{4}\varepsilon_{4}-2r_{1}\varepsilon_{1}+\varepsilon_{4}^{2}-\varepsilon_{1}^{2}\right).$$

Referring to (22),

(28) $$\begin{array}{l} \Delta p^{T}\Delta p=\frac{a_{2}^{2}m^{T}M_{3}^{T}M_{3}m}{{\left(n^{T}m\right)}^{2}}+\frac{a_{2}a_{3}m^{T}M_{3}^{T}M_{2}m}{{\left(n^{T}m\right)}^{2}}+\frac{a_{2}a_{4}m^{T}M_{3}^{T}n}{{\left(n^{T}m\right)}^{2}}+\frac{a_{2}a_{3}m^{T}M_{2}^{T}M_{3}m}{{\left(n^{T}m\right)}^{2}}\\ \;\;\;\;+\frac{a_{3}^{2}m^{T}M_{2}^{T}M_{2}m}{{\left(n^{T}m\right)}^{2}}+\frac{a_{3}a_{4}m^{T}M_{2}^{T}n}{{\left(n^{T}m\right)}^{2}}+\frac{a_{2}a_{4}n^{T}M_{3}m}{{\left(n^{T}m\right)}^{2}}+\frac{a_{3}a_{4}n^{T}M_{2}m}{{\left(n^{T}m\right)}^{2}}+\frac{a_{4}^{2}n^{T}n}{{\left(n^{T}m\right)}^{2}} \end{array}.$$

Further derivations in (28) yields

(29) $$\Delta p^{T}\Delta p=\frac{a_{2}^{2}{\left|d\right|}^{2}}{{\left(n^{T}m\right)}^{2}}+\frac{a_{3}^{2}{\left|g\right|}^{2}}{{\left(n^{T}m\right)}^{2}}+\frac{a_{4}^{2}{\left|n\right|}^{2}}{{\left(n^{T}m\right)}^{2}}+2\frac{-a_{2}a_{3}d^{T}g-a_{2}a_{4}d^{T}n+a_{3}a_{4}g^{T}n}{{\left(n^{T}m\right)}^{2}}.$$

Referring to (29), $\left|d\right|\left|g\right|\geq -d^{T}g$, $\left|d\right|\left|n\right|\geq -d^{T}n$ and $\left|g\right|\left|n\right|\geq g^{T}n$. Therefore,

(30) $$-a_{2}a_{3}d^{T}g-a_{2}a_{4}d^{T}n+a_{3}a_{4}g^{T}n\leq \left|a_{2}\right|\left|a_{3}\right|\left|d\right|\left|g\right|+\left|a_{2}\right|\left|a_{4}\right|\left|d\right|\left|n\right|+\left|a_{3}\right|\left|a_{4}\right|\left|g\right|\left|n\right|.$$

Referring to (30), ${\left(\left|a_{2}\right|\left|d\right|-\left|a_{3}\right|\left|g\right|\right)}^{2}=a_{2}^{2}{\left|d\right|}^{2}+a_{3}^{3}{\left|g\right|}^{2}-2\left|a_{2}\right|\left|a_{3}\right|\left|d\right|\left|g\right|\geq 0$ implies that $(a_{2}^{2}{\left|d\right|}^{2}+a_{3}^{2}{\left|g\right|}^{2})/2\geq \left|a_{2}\right|\left|a_{3}\right|\left|d\right|\left|g\right|$. Similarly, $(a_{2}^{2}{\left|d\right|}^{2}+a_{4}^{2}{\left|n\right|}^{2})/2\geq \left|a_{2}\right|\left|a_{4}\right|\left|d\right|\left|n\right|$ and $(a_{3}^{2}{\left|g\right|}^{2}+a_{4}^{2}{\left|n\right|}^{2})/2\geq \left|a_{3}\right|\left|a_{4}\right|\left|g\right|\left|n\right|$. Therefore, (30) can be further written as

(31) $$-a_{2}a_{3}d^{T}g-a_{2}a_{4}d^{T}n+a_{3}a_{4}g^{T}n\leq a_{2}^{2}{\left|d\right|}^{2}+a_{3}^{2}{\left|g\right|}^{2}+a_{4}^{2}{\left|n\right|}^{2}.$$

Substituting (31) into (29) yields

(32) $$\Delta p^{T}\Delta p\leq 3\left(\frac{a_{2}^{2}{\left|d\right|}^{2}}{{\left(n^{T}m\right)}^{2}}+\frac{a_{3}^{2}{\left|g\right|}^{2}}{{\left(n^{T}m\right)}^{2}}+\frac{a_{4}^{2}{\left|n\right|}^{2}}{{\left(n^{T}m\right)}^{2}}\right).$$

Taking the expectation for both sides of (32),

(33) $$E\left(\Delta p^{T}\Delta p\right)\leq 3\left(\frac{E\left(a_{2}^{2}\right){\left|d\right|}^{2}}{{\left(n^{T}m\right)}^{2}}+\frac{E\left(a_{3}^{2}\right){\left|g\right|}^{2}}{{\left(n^{T}m\right)}^{2}}+\frac{E\left(a_{4}^{2}\right){\left|n\right|}^{2}}{{\left(n^{T}m\right)}^{2}}\right).$$

Because d=l×m, g=k×m and n=k×l, ${\left|d\right|}^{2}={\left|l\right|}^{2}{\left|m\right|}^{2}\sin^{2}\alpha$, ${\left|g\right|}^{2}={\left|k\right|}^{2}{\left|m\right|}^{2}\sin^{2}\beta$, and ${\left|n\right|}^{2}={\left|k\right|}^{2}{\left|l\right|}^{2}\sin^{2}\varphi$, where α, β and φ are the angles between vectors l and m, k and m, k and l, respectively, as shown in Figure 2. The inequality in (33) can be rewritten as

(34) $$E\left(\Delta p^{T}\Delta p\right)\leq 3\left(\frac{E\left(a_{2}^{2}\right){\left|l\right|}^{2}{\left|m\right|}^{2}\sin^{2}\alpha }{{\left(n^{T}m\right)}^{2}}+\frac{E\left(a_{3}^{2}\right){\left|k\right|}^{2}{\left|m\right|}^{2}\sin^{2}\beta }{{\left(n^{T}m\right)}^{2}}+\frac{E\left(a_{4}^{2}\right){\left|k\right|}^{2}{\left|l\right|}^{2}\sin^{2}\varphi }{{\left(n^{T}m\right)}^{2}}\right).$$

It follows that

(35) $$E\left(\Delta p^{T}\Delta p\right)\leq 3\left(\frac{E\left(a_{2}^{2}\right){\left|l\right|}^{2}{\left|m\right|}^{2}}{{\left(n^{T}m\right)}^{2}}+\frac{E\left(a_{3}^{2}\right){\left|k\right|}^{2}{\left|m\right|}^{2}}{{\left(n^{T}m\right)}^{2}}+\frac{E\left(a_{4}^{2}\right){\left|k\right|}^{2}{\left|l\right|}^{2}}{{\left(n^{T}m\right)}^{2}}\right).$$

Because $n^{T}m=\left|n\right|\left|m\right|\cos\psi$ and $\left|n\right|=\left|k\right|\left|l\right|\sin\varphi$, $n^{T}m=\left|m\right|\left|k\right|\left|l\right|\sin\varphi \cos\psi$, where ψ is the angle between m and the normal vector n of the plane containing the three anchors $q_{1}$, $q_{2}$, and $q_{3}$. It follows that

(36) $$E(\Delta p^{T}\Delta p)\leq \frac{3}{\sin^{2}\varphi \cos^{2}\psi }\left(\frac{E\left(a_{2}^{2}\right)}{{\left|k\right|}^{2}}+\frac{E\left(a_{3}^{2}\right)}{{\left|l\right|}^{2}}+\frac{E\left(a_{4}^{2}\right)}{{\left|m\right|}^{2}}\right).$$

$E(\Delta p^{T}\Delta p)$ in (36) is the variance of the mobile node’s 3D positioning error. The bound for $E(\Delta p^{T}\Delta p)$ in (36) applies for both the LOS and NLOS conditions. Note that $E(a_{2}^{2})>0$, $E(a_{3}^{2})>0$, and $E(a_{4}^{2})>0$ in (36). Given that the distance between pairs of anchors, $\left|k\right|$, $\left|l\right|$, and $\left|m\right|$ are decided, (36) suggests that arranging the anchor installation positions with φ= 90° and ψ= 0° leads to reasonably small positioning. Moreover, installing the anchors as separated as possible, i.e., ***k***, ***l,*** and ***m*** being as large as possible, results in reasonably small 3D positioning errors. Note that anchors should be installed at the positions so that ***k***, ***l,*** and ***m*** are as large as possible, provided that the radio receiving intensity between the mobile node and the anchor is within the rated range.

### 3.2. 2D Positioning

**Figure 3:**
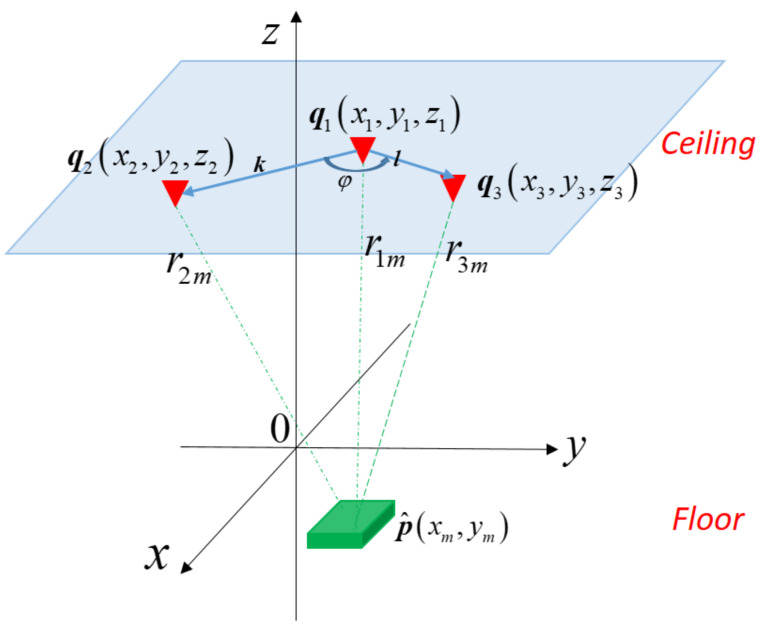
Positioning of a mobile node in a 2D space with three anchors.

Essentially, the mathematical model of 2D positioning error is a special case of the one of 3D positioning error. It takes at least three anchors to determine the 2D position of a mobile node. Denote $q_{i}$ as the vector containing the coordinate $\left(x_{i},y_{i},z_{i}\right)$ of the *i*th anchors, i.e., $q_{i}={\left[x_{i},y_{i},z_{i}\right]}^{T}$, *i* = 1, …, 3. Referring to Figure 3, let $k={\left[x_{2}-x_{1},y_{2}-y_{1},z_{2}-z_{1}\right]}^{T}$ and $l={\left[x_{3}-x_{1},y_{3}-y_{1},z_{3}-z_{1}\right]}^{T}$ be the vectors from the position $q_{1}$ to $q_{2}$ and $q_{1}$ to $q_{3}$, respectively. Compared with Figure 2, the fourth anchor $q_{4}$ in Figure 2 is not needed for 2D positioning as shown in Figure 3. Denote *p*′ and $\hat{p}\prime$ as the vector containing the 2D coordinate the mobile node’s actual position (*x*, *y*) and measured position $\left(x_{m},y_{m}\right)$, respectively, i.e., $\hat{p}\prime ={\left[x,y\right]}^{T}$ and $\hat{p}\prime ={\left[x_{m},y_{m}\right]}^{T}$. Most of the mathematical analysis for 3D positioning in the previous subsection can be used for the analysis of 2D positioning, except that *z* = 0 and $z_{m}=0$.

Setting $z_{m}=0$ in (12) yields

(37) $$\left[\begin{array}{c} \left(r_{1m}^{2}-r_{2m}^{2}\right)\\ \left(r_{1m}^{2}-r_{3m}^{2}\right) \end{array}\right]=\left[\begin{array}{c} 2\left(x_{2}-x_{1}\right)x_{m}+2\left(y_{2}-y_{1}\right)y_{m}+\left(x_{1}^{2}-x_{2}^{2}\right)+\left(y_{1}^{2}-y_{2}^{2}\right)+\left(z_{1}^{2}-z_{2}^{2}\right)\\ 2\left(x_{3}-x_{1}\right)x_{m}+2\left(y_{3}-y_{1}\right)y_{m}+\left(x_{1}^{2}-x_{3}^{2}\right)+\left(y_{1}^{2}-y_{3}^{2}\right)+\left(z_{1}^{2}-z_{3}^{2}\right) \end{array}\right].$$

Therefore,

(38) $$\left[\begin{array}{c} \left(r_{1m}^{2}-r_{2m}^{2})+(x_{2}^{2}-x_{1}^{2})+(y_{2}^{2}-y_{1}^{2})+(z_{2}^{2}-z_{1}^{2}\right)\\ \left(r_{1m}^{2}-r_{3m}^{2})+(x_{3}^{2}-x_{1}^{2})+(y_{3}^{2}-y_{1}^{2})+(z_{3}^{2}-z_{1}^{2}\right) \end{array}\right]=2\left[\begin{array}{cc} \left(x_{2}-x_{1}\right) & \left(y_{2}-y_{1}\right)\\ \left(x_{3}-x_{1}\right) & \left(y_{3}-y_{1}\right) \end{array}\right]\left[\begin{array}{c} x_{m}\\ y_{m} \end{array}\right].$$

Referring to (38), let

(39) $$\Delta r_{m}^{\prime }=\left[\begin{array}{c} \left(r_{1m}^{2}-r_{2m}^{2})+(x_{2}^{2}-x_{1}^{2})+(y_{2}^{2}-y_{1}^{2})+(z_{2}^{2}-z_{1}^{2}\right)\\ \left(r_{1m}^{2}-r_{3m}^{2})+(x_{3}^{2}-x_{1}^{2})+(y_{3}^{2}-y_{1}^{2})+(z_{3}^{2}-z_{1}^{2}\right) \end{array}\right],$$

and

(40) $$H^{\prime }=\left[\begin{array}{cc} \left(x_{2}-x_{1}\right) & \left(y_{2}-y_{1}\right)\\ \left(x_{3}-x_{1}\right) & \left(y_{3}-y_{1}\right) \end{array}\right].$$

Then, (38) is expressed as

(41) $$\Delta r_{m}^{\prime }=2H^{\prime }{\hat{p}}^{\prime },$$

If ***H***′ is nonsingular, i.e., det(***H′***) ≠ 0, the measured position of the mobile node is calculated as

(42) $${\hat{p}}^{\prime }=\frac{1}{2}{\left[H^{\prime }\right]}^{-1}\Delta r_{m}^{\prime }.$$

**Figure 4:**
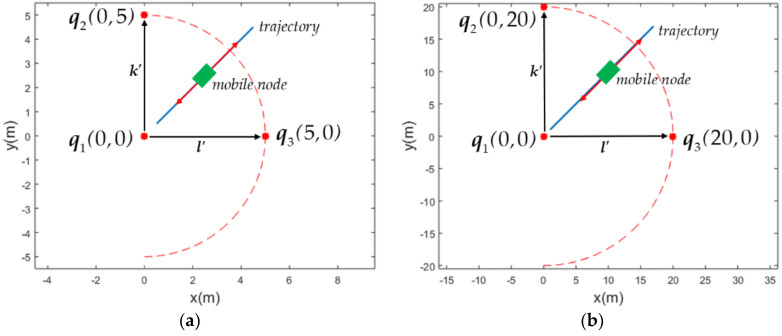
Projection of installation positions of anchors $q_{1},\;q_{2}\;\mathrm{and}\;q_{3}$ onto the X−Y plane and the moving trajectory of the mobile node. (**a**) The environment of 5*m* × 5*m*. (**b**) The environment of 20*m* × 20*m*.

Referring to (42), ${\hat{p}}^{\prime }$ has a deterministic solution under the condition that det(*H*′) ≠ 0. Referring to (40), let $k\prime ={\left[x_{2}-x_{1},y_{2}-y_{1}\right]}^{T}$ and $l\prime ={\left[x_{3}-x_{1},y_{3}-y_{1}\right]}^{T}$. If φ is the angle between vectors $k^{\prime }$ and $l^{\prime }$, $\det\left(H^{\prime }\right)=k\prime \times l\prime =\left|k^{\prime }\right|\left|l^{\prime }\right|\sin\varphi$. Note that ***k***′ and ***l***′ are the projection of ***k*** and ***l*** onto the X-Y plane, respectively, as shown in Figure 4a,b. Therefore, $\hat{p}\prime$ has a deterministic solution provided that φ≠0, i.e., ***k***′ is not parallel with ***l***′.

Referring to (18), the actual position ***p***′ of the mobile node can be theoretically determined similar to the measured position in (42) as

(43) $$p\prime =\frac{1}{2}{\left[H^{\prime }\right]}^{-1}\Delta r^{\prime },$$

where

(44) $$\Delta r^{\prime }=\left[\begin{array}{c} \left(r_{1}^{2}-r_{2}^{2})+(x_{2}^{2}-x_{1}^{2})+(y_{2}^{2}-y_{1}^{2})+(z_{2}^{2}-z_{1}^{2}\right)\\ \left(r_{1}^{2}-r_{3}^{2})+(x_{3}^{2}-x_{1}^{2})+(y_{3}^{2}-y_{1}^{2})+(z_{3}^{2}-z_{1}^{2}\right) \end{array}\right].$$

Denote Δp′ as the mobile node’s positioning error,

(45) $$\Delta p\equiv p^{\prime }-{\hat{p}}^{\prime }=\frac{1}{2}{\left[H^{\prime }\right]}^{-1}\left(\Delta r^{\prime }-\Delta r_{m}^{\prime }\right)=\frac{1}{2}{\left[H^{\prime }\right]}^{-1}\Delta \lambda^{\prime }.$$

where $\Delta \lambda^{\prime }=\Delta r^{\prime }-\Delta r_{m}^{\prime }$ and

(46) $$\Delta \lambda^{\prime }=\left[\begin{array}{c} 2r_{2}\varepsilon_{2}-2r_{1}\varepsilon_{1}+\varepsilon_{2}^{2}-\varepsilon_{1}^{2}\\ 2r_{3}\varepsilon_{3}-2r_{1}\varepsilon_{1}+\varepsilon_{3}^{2}-\varepsilon_{1}^{2} \end{array}\right].$$

Substituting (46) into (45) and performing some derivations yields

(47) $$\Delta p=\frac{a_{2}Ml^{\prime }-a_{3}Mk^{\prime }}{\left|k^{\prime }\right|\left|l^{\prime }\right|\sin\varphi },$$

where $M=\left[\begin{array}{cc} -1 & 0\\ 0 & 1 \end{array}\right]$, $a_{2}$ and $a_{3}$ are as shown in (25) and (26), respectively. Then,

(48) $$\Delta p^{T}\Delta p=\frac{1}{{\left|k^{\prime }\right|}^{2}{\left|l^{\prime }\right|}^{2}\sin^{2}\varphi }\left(a_{2}^{2}{\left|l^{\prime }\right|}^{2}+a_{3}^{2}{\left|k^{\prime }\right|}^{2}-2a_{2}a_{3}{\left[l^{\prime }\right]}^{T}k\prime \right).$$

Referring to (48), $\left|l^{\prime }\right|\left|k^{\prime }\right|\geq -{\left[l^{\prime }\right]}^{T}k^{\prime }$. Therefore,

(49) $$-2a_{2}a_{3}{\left[l^{\prime }\right]}^{T}k\prime \leq 2\left|a_{2}\right|\left|a_{3}\right|\left|l^{\prime }\right|\left|k^{\prime }\right|.$$

Since ${\left(\left|a_{2}\right|\left|l^{\prime }\right|-\left|a_{3}\right|\left|k^{\prime }\right|\right)}^{2}=a_{2}^{2}{\left|l^{\prime }\right|}^{2}+a_{3}^{2}{\left|k^{\prime }\right|}^{2}-2\left|a_{2}\right|\left|a_{3}\right|\left|l^{\prime }\right|\left|k^{\prime }\right|\geq 0$, it implies that $a_{2}^{2}{\left|l^{\prime }\right|}^{2}+a_{3}^{2}{\left|k^{\prime }\right|}^{2}\geq 2\left|a_{2}\right|\left|a_{3}\right|\left|l^{\prime }\right|\left|k^{\prime }\right|$. The inequality (49) can be further written as

(50) $$-2a_{2}a_{3}{\left[l^{\prime }\right]}^{T}k\prime \leq a_{2}^{2}{\left|l^{\prime }\right|}^{2}+a_{3}^{2}{\left|k^{\prime }\right|}^{2}.$$

Substituting (50) into (48) yields

(51) $$\Delta p^{T}\Delta p\leq \frac{2}{{\left|k^{\prime }\right|}^{2}{\left|l^{\prime }\right|}^{2}\sin^{2}\varphi }\left(a_{2}^{2}{\left|l^{\prime }\right|}^{2}+a_{3}^{2}{\left|k^{\prime }\right|}^{2}\right).$$

Taking the expectation for both sides of (51),

(52) $$E\left(\Delta p^{T}\Delta p\right)\leq \frac{2}{{\left|k^{\prime }\right|}^{2}{\left|l^{\prime }\right|}^{2}\sin^{2}\varphi }\left(E\left(a_{2}^{2}\right){\left|l^{\prime }\right|}^{2}+E\left(a_{3}^{2}\right){\left|k^{\prime }\right|}^{2}\right).$$

It can be further simplified as

(53) $$E\left(\Delta p^{T}\Delta p\right)\leq \frac{2}{\sin^{2}\varphi }\left(\frac{E(a_{2}^{2})}{{\left|k^{\prime }\right|}^{2}}+\frac{E(a_{3}^{2})}{{\left|l^{\prime }\right|}^{2}}\right).$$

Similar to the 3D position error, the variance of the mobile node’s 2D positioning error $E(\Delta p^{T}\Delta p)$ in (53) also applies for both the LOS and NLOS conditions. Given that the distance between pairs of anchors $\left|k^{\prime }\right|$ and $\left|l^{\prime }\right|$ are decided, (53) suggests that arranging the anchor positions with φ=90° leads to reasonably small positioning errors. Moreover, installing the anchors as separated as possible, i.e., ***k***′ and ***l***′ being as large as possible, results in reasonably small 2D positioning errors.

## 4. Computer Simulations

Computer simulations were implemented to simulate the mobile node’s 2D and 3D positioning. The mobile node was designed to move following a 2D and 3D straight line in a 2D and 3D environment, respectively. A total of 8000 positioning samples were calculated in 2D or 3D simulations, respectively. In other words, the sampling frequency was designed as 100 Hz and 80 s of positioning errors were calculated and recorded for 2D and 3D simulations. The RMSPE was calculated based on the *N* samples calculated and recorded at the mobile node. Denote $\Delta \bar{p}$ as the RMSPE and $\Delta p_{i}$ as the *i*th sample of positioning error, *i* = 1, …, *N*. Then,

(54) $$\Delta \bar{p}=\sqrt{\frac{\sum_{i=1}^{N}\Delta p_{i}^{T}\Delta p_{i}}{N}},$$

where *N* = 8000. The mobile node’s RMSPE $\Delta \bar{p}$ defined in (54) is utilized for the simulation.

Both the LOS and NLOS conditions are to be simulated. The distance measurement noise $\varepsilon ∼N\left(\mu_{G},\sigma_{G}^{2}\right)$ for the LOS condition and $\varepsilon ∼ST(z;\mu_{ST},\sigma_{ST}^{2},\delta_{ST},\nu_{ST})$ for the NLOS condition. The parameters of the Gaussian distribution for the LOS condition are set as the mean $\mu_{G}=0$ and the standard deviation $\sigma_{G}=0.1m$. The parameters of the skew t-distribution for the NLOS condition are set as $\mu_{ST}=0.1m$, $\sigma_{ST}=0.3m$, $\delta_{ST}=3$, and ν=4. The variance of 3D position errors defined in (36) can be further simplified if the distance measurement noise $\varepsilon ∼N\left(0,\sigma_{G}^{2}\right)$. The terms $E(a_{2}^{2})$, $E(a_{3}^{2})$, and $E(a_{4}^{2})$ in (36) can be replaced with $E\left(a_{2}^{2}\right)=\sigma_{G}^{2}(r_{1}^{2}+r_{2}^{2})$, $E\left(a_{3}^{2}\right)=\sigma_{G}^{2}(r_{1}^{2}+r_{3}^{2})$, and $E\left(a_{4}^{2}\right)=\sigma_{G}^{2}(r_{1}^{2}+r_{4}^{2})$ for the LOS condition. Similarly, the terms $E(a_{2}^{2})$ and $E(a_{3}^{2})$ in the variance of 2D position errors defined in (53) can also be replaced with $E\left(a_{2}^{2}\right)=\sigma_{G}^{2}(r_{1}^{2}+r_{2}^{2})$ and $E\left(a_{3}^{2}\right)=\sigma_{G}^{2}(r_{1}^{2}+r_{3}^{2})$ for the LOS condition.

### 4.1. 2D positioning Simulation

In order to simulate the variation of $\Delta \bar{p}$ with respect to the angle φ between the vectors *k’* and *l’*, the projection of installation positions of three anchors $q_{1},\;q_{2}\;\mathrm{and}\;q_{3}$ onto the X-Y plane are shown in Figure 4a,b, respectively, for the convenience of illustration. Both Figure 4a,b are essentially similar except that the sizes of the simulation environment are different. As shown in Figure 4a,b, 2 anchors $q_{1}$ and $q_{2}$ were installed at fixed positions (0*m*, 0*m*) and (0*m*, 5*m*), and (0*m*, 0*m*) and (0*m*, 20*m*), respectively. The mobile node moves from (0.5*m*, 0.5*m*) to (4.5*m*, 4.5*m*) in Figure 4a and from (2*m*, 2*m*) to (18*m*, 18*m*) in Figure 4b for four round trips. The anchor $q_{3}$ was simulated to be installed at different positions on the circumference with a radius of 5m and 20m, respectively, in Figure 4a,b, but both centered at (0*m*, 0*m*). The angle φ ranged from 0° to 160° as in Figure 4a,b. The heights of $q_{1},\;q_{2}\;\mathrm{and}\;q_{3}$ on the z-axis were assumed to be 4*m*, 4.2*m*, and 4.1*m*, respectively.

**Figure 5:**
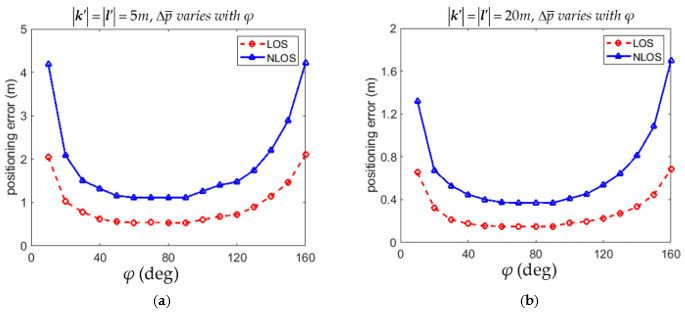
Variation of 2D RMSPE $\Delta \bar{p}$ with angles φ for the LOS and NLOS conditions. (**a**) The environment of 5*m* × 5*m*. (**b**) The environment of 20*m* × 20*m*.

The RMSPE $\Delta \bar{p}$ defined in (54) with different angles of φ for the LOS and NLOS conditions are listed in Table 1 and Table 2, respectively. It is shown in Table 1 that $\Delta \bar{p}$ was minimum if φ = 90° in both 5*m* × 5*m* and of 20*m* × 20*m* environments for the LOS condition. Table 2 shows that $\Delta \bar{p}$ was minimum if φ = 70° in both 5*m* × 5*m* and of 20*m* × 20*m* environments for the NLOS condition. However, $\Delta \bar{p}$ with φ = 90° in both 5*m* × 5*m* and of 20*m* × 20*m* environments were very close to the minimum values occurring at φ = 70°. The variations of $\Delta \bar{p}$ with the angle φ for the LOS and NLOS conditions in both Table 1 and Table 2 are illustrated in Figure 5a,b, respectively. The suggestion based on (53) that the anchor installation arrangement with φ = 90° leads to reasonably small positioning error for both the LOS and NLOS conditions is thus justified from both Table 1 and Table 2.

**Figure 6:**
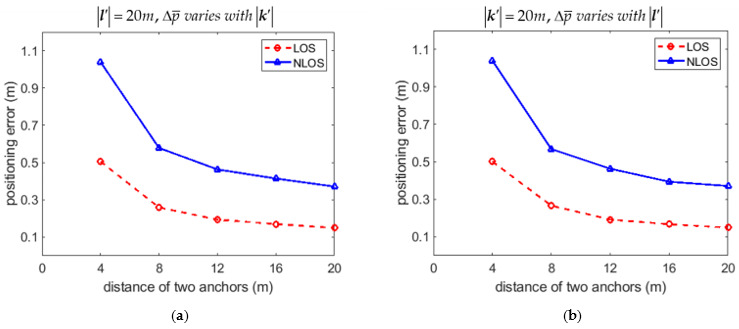
Variation of 2D RMSPE $\Delta \bar{p}$ with different anchor distances $\left|k^{\prime }\right|$ and $\left|l^{\prime }\right|$. (**a**) Variation of $\Delta \bar{p}$ with $\left|k^{\prime }\right|$ provided that $\left|l^{\prime }\right|=20m$; (**b**) Variation of $\Delta \bar{p}$ with $\left|l^{\prime }\right|$ provided that $\left|k^{\prime }\right|=20m$.

The 2D RMSPE $\Delta \bar{p}$ is also affected by the anchor distances $\left|k^{\prime }\right|\;\mathrm{and}\;\left|l^{\prime }\right|$. The variation of $\Delta \bar{p}$ with different $\left|k^{\prime }\right|\;\mathrm{and}\;\left|l^{\prime }\right|$ for the LOS and NLOS conditions are calculated in Table 3 and Table 4, respectively. Both Table 3 and Table 4 show that the 2D RMSPE $\Delta \bar{p}$ decreased as $\left|k^{\prime }\right|$ and/or $\left|l^{\prime }\right|$ increased for both the LOS and NLOS conditions. The variations of $\Delta \bar{p}$ with anchor distances $\left|k^{\prime }\right|\;\mathrm{and}\;\left|l^{\prime }\right|$ for the LOS and NLOS conditions are also illustrated in Figure 6a,b, respectively. The suggestion based on (53) that installing the anchors as separated as possible results in reasonably small positioning error is thus justified.

Since the installation arrangement of anchors suggested in this paper was based on the upper bound of the variance of positioning errors in (53), the accuracy of the upper bound is worth evaluation. Denote the upper bound of variance in (53) for the *i*th sample as $V_{i}^{u}$, then

(55) $$V_{i}^{u}=\frac{2}{\sin^{2}\varphi }\left(\frac{E({\left(a_{2,i}\right)}^{2})}{{\left|k^{\prime }\right|}^{2}}+\frac{E({\left(a_{3,i}^{}\right)}^{2})}{{\left|l^{\prime }\right|}^{2}}\right)$$

where $a_{2,i}$ and $a_{3,i}$ are the parameters $a_{2}$ and $a_{3}$ corresponding to the *i*th sample data. Denote $\Delta {\bar{p}}^{u}$ as the upper bound of average positioning error based on (53) for all *N* samples, then

(56) $$\Delta {\bar{p}}^{u}=\sqrt{\frac{\sum_{i=1}^{N}V_{i}^{u}}{N}}.$$

**Figure 7:**
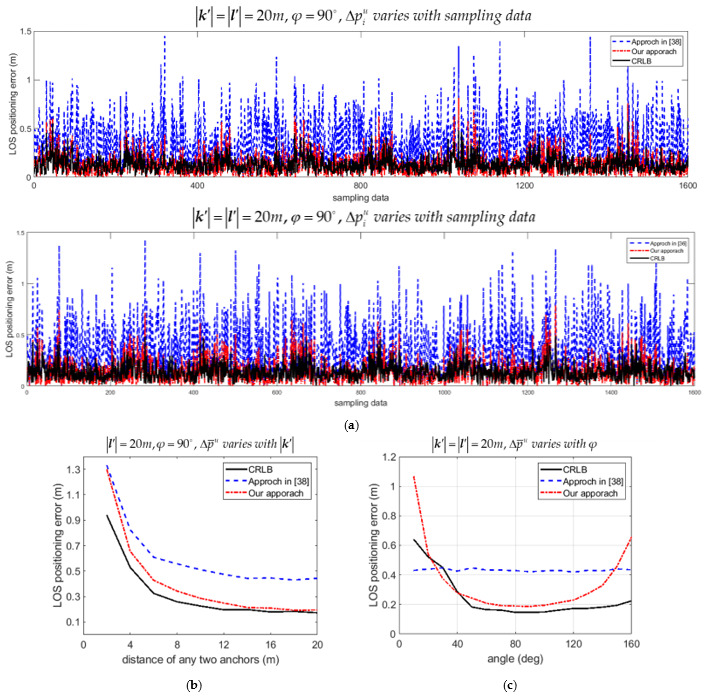
Comparison of upper bound of 2D positioning errors estimated by our approach and the approach in [38] for the LOS condition. (**a**) Variation of $\Delta p_{i}^{u}$ with samples; (**b**) Variation of $\Delta {\bar{p}}^{u}$ with distance $\left|k^{\prime }\right|$; (**c**) Variation of $\Delta {\bar{p}}^{u}$ with angle φ.

**Figure 8:**
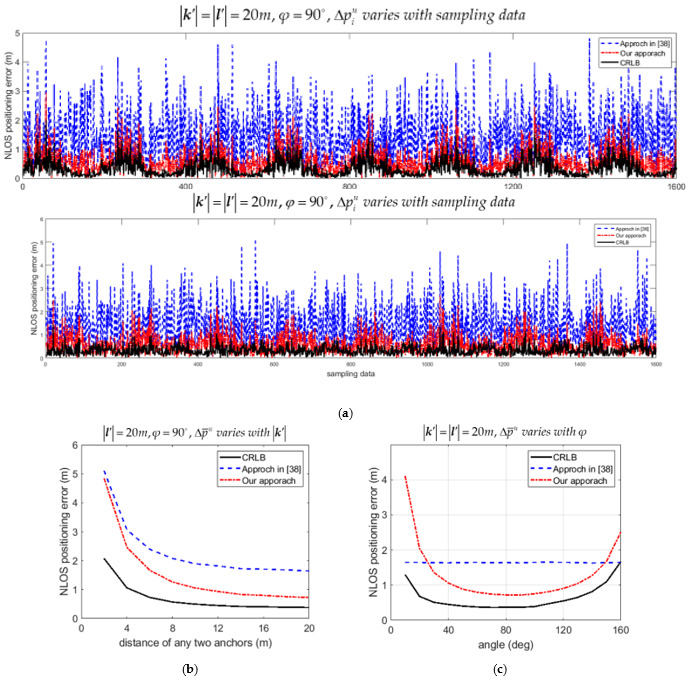
Comparison of upper bound of 2D positioning errors estimated by our approach and the approach in [38] for the NLOS condition. (**a**) Variation of $\Delta p_{i}^{u}$ with samples; (**b**) Variation of $\Delta {\bar{p}}^{u}$ with distance $\left|k^{\prime }\right|$; (**c**) Variation of $\Delta {\bar{p}}^{u}$ with angle φ.

The upper bound of average positioning error $\Delta {\bar{p}}^{u}$ in (56) was compared with the one proposed in [38]. The theoretical Cramer-Rao lower bound (CRLB) was also calculated and plotted for comparison. Let the upper bound of positioning error for the *i*th data $\Delta p_{i}^{u}=\sqrt{V_{i}^{u}}$. The variations of $\Delta p_{i}^{u}$ with samples estimated by our approach and by the approach in [38] are compared in Figure 7a and Figure 8a for the LOS and NLOS condition, respectively. Recall that 8000 samples were recorded in the simulation. Only 20 percent of recorded data are plotted in Figure 7a and Figure 8a for convenience of illustration. Both Figure 7a and Figure 8a show that the upper bound of positioning error $\Delta p_{i}^{u}$ was much lower than the ones calculated by the approach in [38], and much closer to the CRLB for the LOS and NLOS conditions, respectively. The variations of upper bound of average positioning error $\Delta {\bar{p}}^{u}$ with $\left|k^{\prime }\right|$ for the situation that $\left|l^{\prime }\right|$ = 20*m* and φ = 90° were also compared as in Figure 7b and Figure 8b for the LOS and NLOS conditions, respectively. Figure 7c and Figure 8c show the comparison of $\Delta {\bar{p}}^{u}$ with φ if $\left|k^{\prime }\right|$ = $\left|l^{\prime }\right|$ = 20*m* for the LOS and NLOS conditions, respectively. It is shown in Figure 7b,c and Figure 8b,c that $\Delta {\bar{p}}^{u}$ was much lower than the one calculated by the approach in [38], and much closer to the CRLB for both the LOS and NLOS conditions.

### 4.2. 3D Positioning Simulation

**Figure 9:**
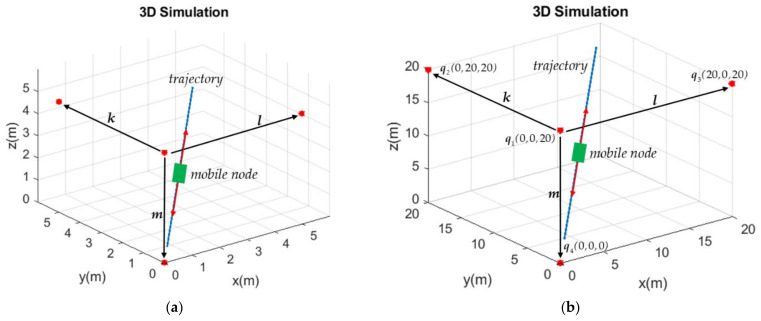
Installation positions of anchors $q_{1},\;q_{2}\;q_{3}\;\mathrm{and}\;q_{4}$ and moving trajectory of the mobile node. (**a**) The environment of 5*m* × 5*m* × 5*m*. (**b**) The environment of 20*m* × 20*m* × 20*m*.

The mobile node’s positioning error defined in (22) was utilized for the 3D simulation. In order to simulate the variations of $\Delta \bar{p}$ with respect to the angles φ between the vectors *k* and *l* and the angle ψ between the vectors *m* and *n*, the anchors $q_{1},\;q_{2}$, $q_{3}$, and $q_{4}$ were assumed to be installed in a smaller environment 5*m* × 5*m* × 5*m* and a larger environment 20*m* × 20*m* × 20*m*, as shown in Figure 9a,b, respectively. The mobile node moves from (0.5*m*, 0.5*m*, 0.5*m*) to (4.5*m*, 4.5 *m*, 4.5*m*) in Figure 9a and from (2*m*, 2*m*, 2*m*) to (18*m*, 18*m*, 18*m*) in Figure 9b for four round trips. The anchor $q_{3}$ and $q_{4}$ were both simulated to be installed at different positions on the circumference with a radius of 5*m* and 20*m*, respectively, in Figure 9a,b, but both centered at the position of $q_{1}$ providing that other anchors were fixed at the positions shown in Figure 9a,b. The positioning errors $\Delta \bar{p}$ were calculated with angles φ and ψ varying from 0° to 160°. Note that φ varies by changing the angle between the vectors *k* and *l* while ψ varies by changing the angle between the vectors ***m*** and ***n***.

**Figure 10:**
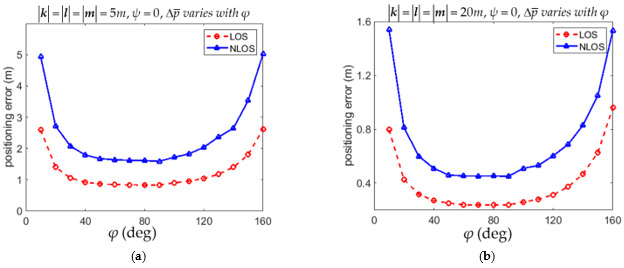
Variation of 3D RMSPE $\Delta \bar{p}$ with angles φ for the LOS and NLOS conditions. (**a**) The environment of 5*m* × 5*m* × 5*m*. (**b**) The environment of 20*m* × 20*m* × 20*m*.

**Figure 11:**
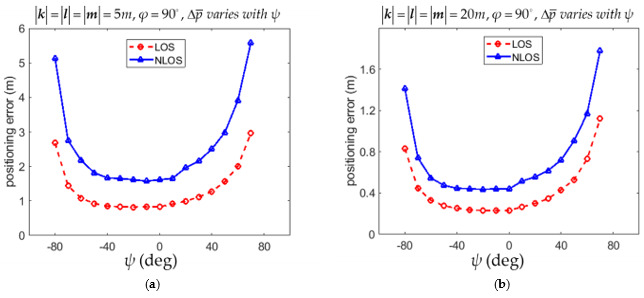
Variation of 3D RMSPE $\Delta \bar{p}$ with angles ψ for the LOS and NLOS conditions. (**a**) The environment of 5*m* × 5*m* × 5*m*. (**b**) The environment of 20*m* × 20*m* × 20*m*.

The RMSPE $\Delta \bar{p}$ defined in (54) with different angles of φ for the LOS and NLOS conditions are listed in Table 5 and Table 6, respectively. It is shown in Table 5 that $\Delta \bar{p}$ was minimum at φ = 80° in the 5*m* × 5*m* × 5*m* environment while $\Delta \bar{p}$ was minimum at φ = 70° in the 20*m* × 20*m* × 20*m* environment for the LOS condition. However, $\Delta \bar{p}$ with φ = 90° in both 5*m* × 5*m* × 5*m* and 20*m* × 20*m* × 20*m* environments were very close to the minimum values occurring at φ = 80° and 70° in the 5*m* × 5*m* × 5*m* and 20*m* × 20*m* × 20*m* environment, respectively. Table 6 shows that $\Delta \bar{p}$ was minimum φ = 90° in both 5*m* × 5*m* × 5*m* and 20*m* × 20*m* × 20*m* environments for the NLOS condition. The variations of $\Delta \bar{p}$ with the angle φ for the LOS and NLOS conditions in both Table 5 and Table 6 are illustrated in Figure 10a,b, respectively. As for the angle ψ, it is shown in Table 7 that $\Delta \bar{p}$ was minimum is minimum at ψ = 20° in both 5*m* × 5*m* × 5*m* and 20*m* × 20*m* × 20*m* environments for the LOS condition. However, $\Delta \bar{p}$ with ψ = 0° in both 5*m* × 5*m* × 5*m* and 20*m* × 20*m* × 20*m* environments were also very close to the minimum values occurring at ψ = 20° in both environments. It is shown in Table 8 that $\Delta \bar{p}$ was minimum at ψ = 10° in the 5*m* × 5*m* × 5*m* environment while $\Delta \bar{p}$ was minimum at φ = 20° in the 20*m* × 20*m* × 20*m* environment for the NLOS condition. However, $\Delta \bar{p}$ with ψ = 0° in both 5*m* × 5*m* × 5*m* and 20*m* × 20*m* × 20*m* environments were very close to the minimum values occurring at ψ = 10° and 20° in the 5*m* × 5*m* × 5*m* and 20*m* × 20*m* × 20*m* environment, respectively. The variations of $\Delta \bar{p}$ with the angle ψ for the LOS and NLOS conditions in both Table 7 and Table 8 are illustrated in Figure 11a and Figure 11b, respectively. The suggestion based on (36) that the anchor installation arrangement with φ = 90° and ψ = 0° leads to reasonably small positioning errors for both the LOS and NLOS conditions is thus justified from Table 5, Table 6, Table 7 and Table 8.

## 5. Experiments

**Figure 12:**
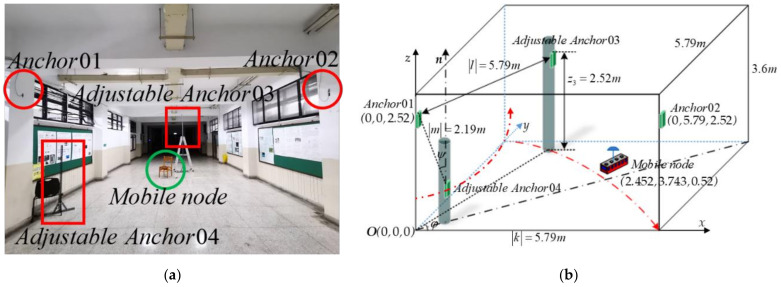
Experiment of measuring mobile node’s positioning errors. (**a**) Photograph showing the positions of the four anchors and the mobile node for the LOS condition; (**b**) Illustration of the experimental environment and the moving trajectory of the adjustable anchors.

After the computer simulations shown in the previous section, an experiment was conducted to further verify the results obtained in 3D positioning for both the LOS and NLOS conditions. The 3D positioning experiment for the LOS condition was conducted in the environment where no obstacle was placed between the mobile node and every anchor. The experiment was implemented in a corridor outside of the laboratory in an environment 6*m* × 6*m* × 3.6*m*. The installation positions of the four anchors are shown in Figure 12a, where anchors $q_{1}$ and $q_{2}$ were installed on the wall with fixed positions. In contrast, the positions of $q_{3}$ and $q_{4}$ are adjustable for the positioning evaluations with different angles φ and ψ. The mobile node moves in the experiment environment with 0.52m above the ground. The illustration of the experiment environment and the moving trajectories of adjustable anchors $q_{3}$ and $q_{4}$ are shown in Figure 12b. The mobile node calculates the 3D positioning with EKF [45] and records the positions with a sampling frequency of 100 Hz. The RMSPEs $\Delta \bar{p}$ were calculated for every 600 samples.

In the first LOS experiment, the anchors $q_{1}$ and $q_{2}$ were installed at the positions with coordinates ${\left[0,0,2.52\right]}^{T}$ and ${\left[0,5.79,2.52\right]}^{T}$ respectively, as shown in Figure 12b. It resulted in $\left|k\right|$ being equal to 5.79*m*. The installation positions of anchors $q_{3}$ were adjusted following the trajectory shown in Figure 12b with φ varying from 10° to 90°. Note that the z coordinate of $q_{3}$ was kept constant, 2.52*m,* while changing the installation positions. Therefore, $\left|l\right|\;=$ 5.79*m*. The installation position of $q_{4}$ was also adjusted following the trajectory shown in Figure 12b with ψ varying from 0° to 65°. It is shown in Figure 12b that $\left|m\right|$ remained a constant, 2.49*m*, while ψ changed from 0° to 65°. The mobile node was parked at the position ${\left[2.45,\;3.74,\;0.52\right]}^{T}$ to measure the mobile node’s 3D position for different anchor positions. The RMSPEs $\Delta \bar{p}$ were calculated with φ varying from 10° to 90° and yet ψ was set to be 0°. Similarly, the RMSPEs $\Delta \bar{p}$ were calculated with ψ ranging from 0° to 65° and yet φ was set to be 90°. The RMSPEs $\Delta \bar{p}$ corresponding to different combinations of φ and ψ are shown in Table 9. Table 9 shows that $\Delta \bar{p}$ was minimum if the installation position of $q_{3}$ is adjusted so that φ = 90° under the condition ψ = 0°; or if the installation of $q_{4}$ is adjusted so that ψ = 0° under the condition φ = 90°.

In the second LOS experiment, the mobile node was still parked at the position ${\left[2.45,\;3.74,\;0.52\right]}^{T}$, and both $q_{3}$ and $q_{4}$ were set at the position so that φ = 90° and ψ = 0°. The RMSPEs $\Delta \bar{p}$ were measured and calculated while changing both $\left|k\right|$ and $\left|l\right|$ from 1.79*m* to 5.79*m*, and changing $\left|m\right|$ from 1.19*m* to 2.19*m*. Note that $\Delta \bar{p}$ was calculated by changing only one length of vector at a time with the lengths of two other vectors remaining constants. The experiment results are shown in Table 10. Table 10 shows that the $\Delta \bar{p}$ decreased if the lengths of 3 vectors $\left|k\right|$, $\left|l\right|$, and $\left|m\right|$ were appropriately increased.

**Figure 13:**
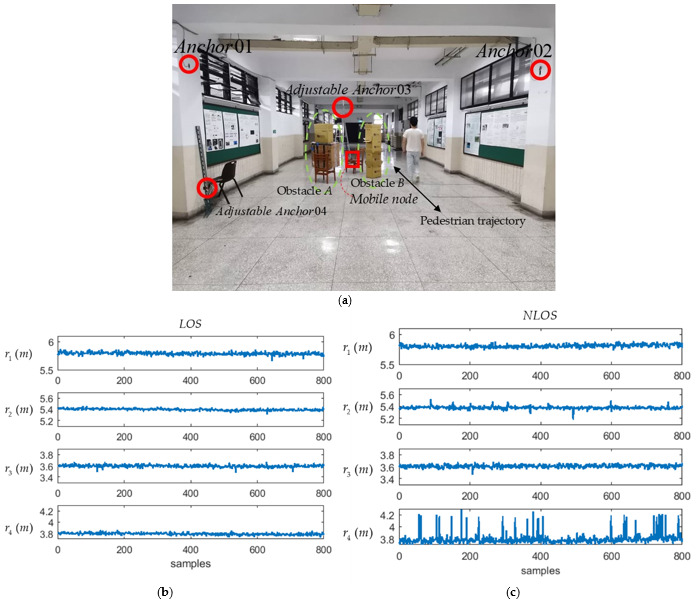
Experiment of measuring the mobile node’s positioning errors. (**a**) Photograph showing the positions of the four anchors and obstacles; (**b**) Measured distances between the mobile node and four anchors for the LOS condition; (**c**) Measured distances between the mobile node and four anchors for the NLOS condition.

The 3D positioning experiment for the NLOS condition was conducted in the same environment as in the LOS experiment, except that several stationary and moving obstacles were placed between the mobile node and the anchors. Figure 13a shows that a stationary obstacle A with the dimension 0.7*m* × 0.4*m* × 1.65*m* was placed between the mobile node and the anchor $q_{4}$ while a stationary obstacle B with the dimension 0.6*m* × 0.25*m* × 1.65*m* was placed between the mobile node and the anchor $q_{2}$. In addition to the stationary obstacles, a pedestrian was arranged to walk back and forth following a straight line between the mobile node and the anchor $q_{2}$ shown in Figure 13a. The measured distance $r_{i}$ between the mobile node and every *i*th anchor $q_{i}$, *i* = 1, …, 4, for the LOS and NLOS conditions are compared in Figure 13b and Figure 13c, respectively. From the measured distances $r_{1},\ldots ,r_{4}$ compared in Figure 13b,c, it is shown that both stationary and moving obstacles did affect the distance measurement. The same 3D positioning experiment for the NLOS condition was conducted as for the LOS condition. The RMSPEs $\Delta \bar{p}$ corresponding to different combinations of φ and ψ for the NLOS condition are shown in Table 11. Table 11 shows that $\Delta \bar{p}$ was minimum if the installation position of $q_{3}$ is adjusted so that φ = 90° under the condition ψ = 0°. The RMSPE $\Delta \bar{p}$ was also minimum if the installation of $q_{4}$ is adjusted so that ψ = 0° under the condition φ = 90°. The RMSPEs $\Delta \bar{p}$ were calculated by changing only one vector length at a time with the lengths of two other vectors remaining constants. The experiment results are shown in Table 12. Table 12 shows that the $\Delta \bar{p}$ decreased if the lengths of three vectors $\left|k\right|$, $\left|l\right|$, and $\left|m\right|$ were appropriately increased.

The suggestion based on (36) that the anchor installation arrangement with φ = 90° and ψ = 0° leads to reasonably small 3D positioning errors for both the LOS and NLOS conditions is thus justified from the experimental results in Table 9 and Table 11. Moreover, the suggestion that installing the anchors as separated as possible leads to reasonably small 3D positioning errors is also justified from the experimental results in Table 10 and Table 12.

## 6. Conclusions

The upper bounds of variance of both 2D and 3D positioning errors were derived in this paper. The mathematical model for the variance of positioning errors shed light on some tips regarding installation positions of anchors. The positioning errors can be reduced through an appropriate arrangement of anchors’ installation positions according to the mathematical model derived in the paper. Computer simulations and practical experiments were conducted to verify the suggestions of anchor installation positions obtained from the mathematical model.

The positioning accuracy of UWB sensors has attracted a lot of attention lately due to their cost-effective positioning performance. The positioning errors analyzed in the paper were limited to the 2D or 3D positions only. However, the positioning errors can be expanded to include the linear and angular velocities and accelerations if the UWB sensors are combined with the inertial measurement units (IMU). Further works can be performed to derive the mathematical model for the “expanded” positioning errors so that more installation suggestions regarding improving positioning accuracy can be obtained from the model. Further works can also be performed to derive the lower bound of variance of positioning errors based on the proposed mathematical model. The range of positioning errors associated with a combination of anchor installation positions can be estimated with more accuracy if both upper and lower bounds of positioning errors are calculated.

## Figures and Tables

**Table 1:** The 2D RMSPE $\Delta \bar{p}$ (m) with varying φ in the 5*m* × 5*m* and 20*m* × 20*m* environments for the LOS condition.

φ (deg)	10	20	30	40	50	60	70	80	90	100	110	120	130	140	150	160
5*m* × 5*m*	2.044	1.022	0.783	0.620	0.556	0.539	0.540	0.536	**0.526**	0.606	0.677	0.723	0.891	1.148	1.458	2.104
20*m* × 20*m*	0.654	0.325	0.213	0.176	0.155	0.150	0.150	0.149	**0.149**	0.184	0.195	0.227	0.270	0.333	0.448	0.684

**Table 2:** The 2D RMSPE $\Delta \bar{p}$ (m) with varying φ in the 5*m* × 5*m* and 20*m* × 20*m* environments for the NLOS condition.

φ (deg)	10	20	30	40	50	60	70	80	90	100	110	120	130	140	150	160
5*m* × 5*m*	4.192	2.091	1.513	1.323	1.155	1.117	**1.112**	1.113	**1.114**	1.261	1.402	1.477	1.745	2.210	2.891	4.219
20*m* × 20*m*	1.323	0.675	0.530	0.448	0.402	0.375	**0.368**	0.373	**0.371**	0.412	0.453	0.538	0.644	0.813	1.085	1.699

**Table 3:** The 2D RMSPE $\Delta \bar{p}$ (m) with varying anchor distances $\left|k\right|$ and $\left|l\right|$ for the LOS condition.

Distances (m)	4	8	12	16	20
|***k***’| (m)	0.505	0.259	0.194	0.170	**0.149**
|***l***’| (m)	0.501	0.266	0.192	0.167	**0.149**

**Table 4:** The 2D RMSPE $\Delta \bar{p}$ (m) with varying anchor distances $\left|k\right|$ and $\left|l\right|$ for the NLOS condition.

Distances (m)	4	8	12	16	20
|***k***’| (m)	1.039	0.578	0.463	0.414	**0.371**
|***l***’| (m)	1.040	0.568	0.463	0.394	**0.371**

**Table 5:** The 3D RMSPE $\Delta \bar{p}$ (m) with varying φ in the 5*m* × 5*m* × 5*m* and 20*m* × 20*m* × 20*m* environments for the LOS condition.

φ (deg)	10	20	30	40	50	60	70	80	90	100	110	120	130	140	150	160
(5*m*)^3^	2.593	1.403	1.058	0.923	0.872	0.847	0.838	**0.835**	**0.837**	0.903	0.957	1.043	1.177	1.400	1.805	2.622
(20*m*)^3^	0.797	0.427	0.318	0.270	0.252	0.240	**0.238**	0.239	**0.239**	0.259	0.281	0.313	0.373	0.467	0.627	0.961

**Table 6:** The 3D RMSPE $\Delta \bar{p}$ (m) with varying φ in the 5*m* × 5*m* × 5*m* and 20*m* × 20*m* × 20*m* environments for the NLOS condition.

φ (deg)	10	20	30	40	50	60	70	80	90	100	110	120	130	140	150	160
(5*m*)^3^	4.936	2.725	2.076	1.794	1.676	1.639	1.621	1.614	**1.591**	1.719	1.828	2.039	2.368	2.649	3.535	5.021
(20*m*)^3^	1.539	0.813	0.599	0.510	0.460	0.454	0.452	0.454	**0.450**	0.510	0.531	0.603	0.687	0.830	1.050	1.533

**Table 7:** The 3D RMSPE $\Delta \bar{p}$ (m) with varying ψ in the 5*m* × 5*m* × 5*m* and 20*m* × 20*m* × 20*m* environments for the LOS condition.

ψ (deg)	80	70	60	50	40	30	20	10	0	−10	−20	−30	−40	−50	−60	−70
(5*m*)^3^	2.679	1.437	1.082	0.925	0.848	0.830	**0.822**	0.834	**0.828**	0.910	0.994	1.113	1.274	1.559	2.002	2.967
(20*m*)^3^	0.830	0.446	0.330	0.275	0.252	0.237	**0.231**	0.231	**0.232**	0.265	0.299	0.345	0.431	0.529	0.729	1.120

**Table 8:** The 3D RMSPE $\Delta \bar{p}$ (m) with varying ψ in the 5*m* × 5*m* × 5*m* and 20*m* × 20*m* × 20*m* environments for the NLOS condition.

ψ (deg)	80	70	60	50	40	30	20	10	0	−10	−20	−30	−40	−50	−60	−70
(5*m*)^3^	5.139	2.750	2.174	1.817	1.667	1.650	1.615	**1.575**	**1.614**	1.655	1.962	2.156	2.502	2.985	3.911	5.593
(20*m*)^3^	1.415	0.742	0.547	0.474	0.446	0.441	**0.435**	0.441	**0.438**	0.516	0.556	0.618	0.720	0.906	1.169	1.780

**Table 9:** The 3D RMSPE $\Delta \bar{p}$ with varying angles φ and ψ for the LOS condition.

	φ (deg)	ψ (deg)	$\Delta \bar{p}\;(m)$
group 1	90	0	**0.0743**
	70	0	0.1366
	55	0	0.2214
	40	0	0.2965
	25	0	0.6745
	10	0	1.4545
group 2	90	0	**0.0743**
	90	20	0.1404
	90	35	0.2136
	90	50	0.3018
	90	65	0.5648

**Table 10:** The 3D RMSPE $\Delta \bar{p}$ with varying anchor distances $\left|k\right|$, $\left|l\right|$, and $\left|m\right|$ for the LOS condition.

	|*k*| (*m*)	|*l*| (*m*)	|*m*| (*m*)	$\Delta \bar{p}\;(m)$
group 1	5.79	5.79	2.19	**0.0743**
	5.79	4.79	2.19	0.1115
	5.79	3.79	2.19	0.1218
	5.79	2.79	2.19	0.1807
	5.79	1.79	2.19	0.3169
group 2	5.79	5.79	2.19	**0.0743**
	5.79	5.79	1.69	0.1850
	5.79	5.79	1.19	0.2852
group 3	5.79	5.79	2.19	**0.0743**
	4.79	5.79	2.19	0.1239
	3.79	5.79	2.19	0.1544
	2.79	5.79	2.19	0.1879
	1.79	5.79	2.19	0.2989

**Table 11:** The 3D RMSPE $\Delta \bar{p}$ environments for the NLOS condition. φ and ψ for the NLOS condition.

	φ (deg)	ψ (deg)	$\Delta \bar{p}\;(m)$
group 1	90	0	**0.5452**
	70	0	0.6355
	55	0	0.6758
	40	0	0.9418
	25	0	1.6660
	10	0	2.9914
group 2	90	0	**0.5452**
	90	20	0.5659
	90	35	0.7983
	90	50	1.0277
	90	65	1.1303

**Table 12:** The 3D RMSPE $\Delta \bar{p}$ with varying anchor distances $\left|k\right|$, $\left|l\right|$, and $\left|m\right|$ for the NLOS condition.

	|*k*| (*m*)	|*l*| (*m*)	|*m*| (*m*)	$\Delta \bar{p}\;(m)$
group 1	5.79	5.79	2.19	**0.5452**
	5.79	4.79	2.19	0.7263
	5.79	3.79	2.19	0.7575
	5.79	2.79	2.19	0.8255
	5.79	1.79	2.19	1.0081
group 2	5.79	5.79	2.19	**0.5452**
	5.79	5.79	1.69	0.8880
	5.79	5.79	1.19	1.0658
group 3	5.79	5.79	2.19	**0.5452**
	4.79	5.79	2.19	0.7302
	3.79	5.79	2.19	0.7333
	2.79	5.79	2.19	0.7593
	1.79	5.79	2.19	0.9898
